# To Avoid Infection

**Published:** 1881-07

**Authors:** 


### TO AVOID INFECTION.

Dr. Littlejohn, Medical Officer of Health, Glasgow, Scotland, gives the following rules to ensure immunity, as far as possible, from infection, by those who are obliged to visit or attend upon the sick, as physicians, nurses, &c.:

1. Never to visit the houses of the sick—fasting.

2. To insist that the ventilation of the apartment to be entered is as free as possible. For this purpose, during the visit, the window must be raised, and the door kept open. Such a procedure exposes the patient to no risk; and, at the same time, the special poison of infection is so diluted as to be practically innocuous.

3. To remain in the presence of the sick no longer than is necessary.

4. Afterwards, free exposure to the open air sufficiently disinfects the clothing; but during epidemics, when there is a large amount of sickness, the clothes worn during the day should be exchanged for others before mixing freely with other persons.

5. And, in these circumstances, free ablution of the hands, aided by the use of such a disinfectant as Condy's fluid, is absolutely necessary.

(It is advisible, too, for the attendant, when in the sick room, to keep on that side of the patient from which any current of air comes; as from an open window, so that the current shall be from attendant, to patient, and not from patient to attendant—Ed. C. H. C.)—*Canada Health Journal*

